# The development of items for a physiotherapy clinical programme evaluation tool

**DOI:** 10.4102/sajp.v79i1.1908

**Published:** 2023-11-14

**Authors:** Vaneshveri Naidoo, Aimee V. Stewart, Morake E. Douglas Maleka

**Affiliations:** 1 Department of Physiotherapy, Faculty of Health Sciences, University of the Witwatersrand, Johannesburg, South Africa; 2 Department of Physiotherapy, Faculty of Health Care Sciences, Sefako Makgato Health Sciences University, Pretoria, South Africa

**Keywords:** physiotherapy, clinical education, evaluation, focus group discussion, qualitative

## Abstract

**Background:**

Evaluating a physiotherapy clinical education programme is challenging, given its complex and multidimensional nature, resulting in a paucity of research on the topic.

**Objectives:**

The objective of our study, which was part of a larger study, was to identify items that could be included in a tool to evaluate a physiotherapy clinical education programme in South Africa.

**Method:**

A qualitative study utilising focused group discussions including academics, clinical educators and clinicians was undertaken. A broad script that delved into clinical education experience was used. An inductive thematic content analysis using MaxQda version 2018.2 was undertaken; the data were coded, and similar foci were categorised and subcategorised. This process led to the identification of themes. Both triangulation of the data (member checks, field note comparison, observer reflection and verification of the data) and assuring the data’s trustworthiness (credibility, dependability and confirmability) were undertaken.

**Results:**

Fourteen focus group discussions were held countrywide. Three themes emerged from the data. A macro theme included all governance issues, a meso theme included all structural issues and a micro theme included all aspects related to clinical experience.

**Conclusion:**

The complex nature of clinical physiotherapy education and its diversity can be seen in these emerging themes. All the categories and subcategories making up these themes must now be considered in the next step of developing this tool.

**Clinical implications:**

Quality assurance and minimal training standards may be ensured.

## Introduction

Over a prolonged period, scholars have attempted to define and measure quality in physiotherapy clinical education. This has been a complicated process because of the complex, multidimensional nature of clinical education and the paucity of research (Higgs 1993; Jette et al. 2014; McCallum et al. 2013; Stachura, Garven & Reed 2000; Strohschein, Hagler & May 2002). A consensus has yet to be reached on what may be alluded to as the quality of clinical physiotherapy education. The quality of clinical education appears to be affected by the *processes* involved in clinical education, namely models of teaching and learning that lead to the development of a competent graduate, the enhancement of technical and non-technical skills, the attitudes and behaviour of students and the roles and relationships between students and clinical educators (Dunfee 2008; Jette et al. 2014; McCallum et al. 2013; Moghadam, Kashfe & Abdi 2017; Stachura et al. 2000; Strohschein et al. 2002).

Because of the challenge experienced in measuring quality in clinical education, Strohschein et al. (2002) advocated for clear clinical education goals and objectives, as had Higgs (1993) and Stachura et al. (2000) in earlier papers. These clear clinical education goals and objectives should facilitate appropriate processes and models of clinical education. They recommended conducting more research to identify prominent features of current clinical education processes using qualitative and quantitative research methods. They suggested that a tool(s) must be developed to measure the important aspects of physiotherapy clinical education.

The quality of clinical education can only be defined and measured if standardisation in structure, processes and outcomes exists in physiotherapy programmes; structure – standardisation of the number and duration of clinical education experiences; standardised forms for communication of important information with clinical sites or standardised supervisory models that allow for student autonomy over time and the use of a standardised national student evaluation form and standardised tools to assess stakeholder satisfaction with clinical education (Jette et al. 2014).

It seems that a lack of standardisation in the structure and processes of clinical education, more so than outcomes, impacts the ability to define quality and evaluate physiotherapy clinical education. Furthermore, the need for more consensus regarding what to measure and how to measure components of structure, process and outcome and the lack of a reliable measuring tool(s) amplifies this problem. Holistic evaluation of clinical education must examine the structure, process and outcome using a valid and reliable tool (standardised measure). Thus, our study aimed to identify items that could be included in a tool to evaluate clinical physiotherapy education in South Africa.

## Methods

This qualitative study involving focus group discussions (FGDs) was undertaken nationwide in South Africa. Permission to undertake the study at the universities where physiotherapists are trained was obtained from various clinical sites. All participants signed informed consent before being included in our study.

Purposive sampling of participants included academics from seven of the eight South African universities involved in educating physiotherapy students, physiotherapy clinicians, physiotherapy clinical managers, physiotherapy clinical educators and community service physiotherapists from various clinical sites. The FGDs included either mixed groups of academics and clinicians or separate academic and clinician groups, depending on the availability of participants.

Before conducting the FGDs, the first author undertook a pilot study of two FGDs to ensure that she ran the FGDs in a manner that would provide participants discussed issues in a safe environment and that she asked appropriate open-ended questions like ‘tell me about your clinical education experience’ with suitable prompts as needed. This pilot study was conducted with colleagues in the Physiotherapy Department of University of the Witwatersrand and a senior academic observer who discussed the FGD procedure with the first author after each pilot FGD. She then conducted all the FGDs countrywide.

The FGDs included 6–8 participants, and each discussion lasted approximately 2 h with appropriate comfort breaks and refreshments. The FGDs were audio-recorded, and the collected demographic information was kept separately from the audio recordings. The first author kept field notes during the FGDs.

The data were analysed using Tesch’s (1992) method of data analysis for qualitative studies (Vuso & James 2017). The audio recordings were transcribed verbatim by a professional transcriber. The first author then cross-checked the transcriptions with the audio recordings to minimise any errors that may have occurred during the transcription. The transcripts were then sent to the FGD participants for verification and were subsequently confirmed as an accurate account of the discussions.

An inductive thematic content analysis using MaxQda version 2018.2 was undertaken; the data were coded, and similar foci were categorised and subcategorised. This process led to the identification of themes (Greenwood et al. 2017; Vuso & James 2017). Two authors (A.S. and D.M.) and an independent researcher checked the thematic content analysis on a selection of transcripts. There was a high degree of agreement, and where there were minor differences, these were discussed and resolved leading to an agreed list of codes, categories and subcategories. They then identified themes from the codes and categories discussed them with the first author and reached a consensus on any minor differences.

Thus, data triangulation was assured through member checks, field note comparison, observer reflection and the above data verification. This triangulation aimed to ‘overcome the inherent biases in a single investigator, single theory or single method approach’ (Halcomb & Andrew 2005).

To ensure trustworthiness that is the credibility, dependability, confirmability and transferability of the data, the following steps were undertaken.

Prolonged interaction with participants during the FGDs; audio recordings and transcriptions; conferring with the observer of the pilot FGDs; using an interview guide during the FGDs; checking the transcripts and comparing them to the field notes taken during the FGDs; member checking and checking of codes, categories and themes by three researchers. Transferability was assured by purposively choosing the sample, namely the academics in the training universities, clinicians and clinical educators involved in the clinical training of undergraduate physiotherapy students (Connelly 2016; Krefting 1991).

The first author considered her background and perceptions in collecting the data during a reflective process (Krefting 1991). She currently coordinates the clinical training programme at the University of the Witwatersrand. Before this, she was a physiotherapy clinician.

### Ethical consideration

Ethical clearance to conduct this study was obtained from the University of the Witwatersrand, Human Research Ethics Committee (M210160). A second ethics cleareance certificate was applied for as the first one (M140706) expired.

## Results

Fourteen FGDs were held countrywide, including seven of the eight universities and three clinical physiotherapy departments. Eighty-one participants were included with approximately eight participants per group, either in groups including academics and clinicians or just academics and clinicians. Data saturation was obtained after eight FGDs. However, the first author continued collecting data for 14 FGDs given that the participants had been invited to participate in the study 6–12 months before the data collection to accommodate academic timetables and clinician responsibilities (see Table 1).

**TABLE 1:** Demographic profile of study participants (*n* = 80).

Age Mean	Age s.d.	Qualification obtained				Gender		Number of years qualified		Type of employment			Job title†											Number of years pilot participants involved in academia(*n* = 11)	
		
		BPT	BSc	MSc	PhD	M	F	*n*	s.d.	Academia	Pvt practice	DoH	Lecturer	Snr lecturer	Associate professor	Clinical educator	Clinical manager	Clinician			Other			Mean	s.d.

																		CS	Jnr	Snr	Dep Dean	Clin Coord.	Tutor		
35.5	±2.8	3	47	18	12	9	71	13.7	±7.25	50.5	1.5	28	30	6	3	6.5	2	11	9	3	1	3	1	7	±6.2

BPT, Bachelor of Physiotherapy; BSc, Bachelor of Science in Physiotherapy; MSc, Master of Science in Physiotherapy; PhD, Doctor of Philosophy; Pvt Practice, private practice; DoH, Department of Health; Snr lecturer, senior lecturer; CS, community service physiotherapist; Jnr, junior physiotherapist; Snr, senior physiotherapist; Dep Dean, deputy dean; Clin Coord, clinical coordinator; M, male; F, female; s.d., standard deviation.

† , Missing data therefore no’s do not tally to 80.

**FIGURE 1:**
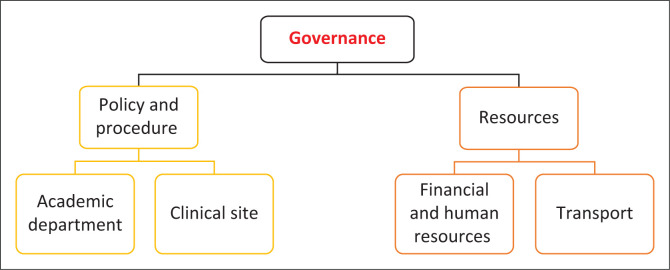
Macro level – governance.

**FIGURE 2:**
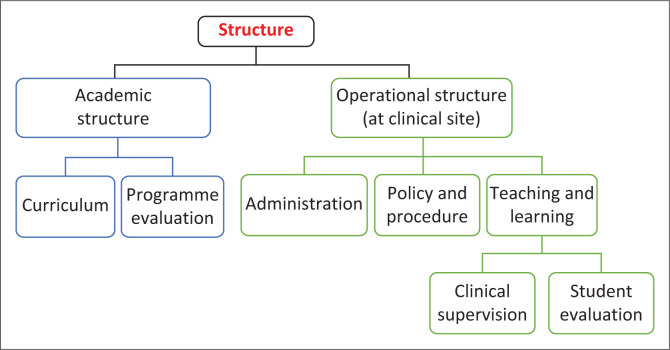
Meso level – structure.

**FIGURE 3:**
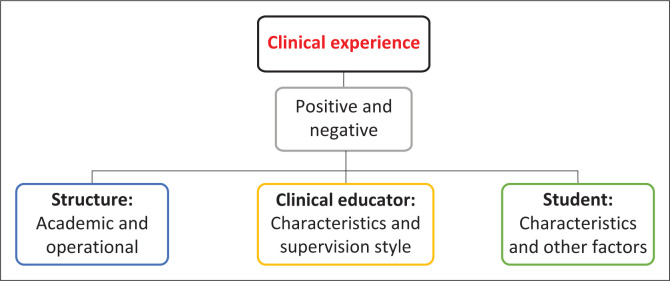
Micro level – clinical experience.

Governance, structure and clinical experience emerged as themes from the inductive thematic content analysis. These themes are macro-, meso- and microstructures (see Figure 1, Figure 2 and Figure 3). The macro level includes the policies, procedures and systems; the meso level is the organisational structure of the academic department and clinical site and the micro level relates to the student’s clinical learning experience. The items of the tool were generated from the resultant codes of each section. A preliminary tool of 131 questions and three sections was developed: *governance* contained 17 questions; *academic structure* contained 55 questions and *operational structure* contained 59 questions.

## Discussion

The 14 FGDs yielded three themes related to clinical physiotherapy education namely governance (macro), structure (meso) and clinical experience (micro). The complexity and diversity of physiotherapy clinical education both in South Africa and in the global community can be seen in these themes developed from the collected data (Higgs 1993; Jette et al. 2014; McCallum et al. 2013; Patton, Higgs & Smith 2018; Stachura et al. 2000; Strohschein et al. 2002). To identify items to be included in a tool to evaluate physiotherapy clinical education, all the categories and subcategories making up these broad themes need to be considered to ensure that a tool developed to evaluate physiotherapy clinical education is comprehensive and sufficiently detailed.

### Governance

Governance (macro level) is defined as the ‘rules for collective action and decision-making in a system with diverse players and organisations’ (Pyone, Smith & Van Den Broek 2017). In physiotherapy clinical education, governance pertains to the policies and procedures that must be followed to ensure that clinical physiotherapy education meets the minimum quality and standard requirements (Ferlie, Musselin & Andresani 2008). In South Africa, these include higher education institutional policies (national and within the institutions), the Health Professions Council of South Africa, the Department of Health and World Physiotherapy. This alignment will ensure that minimal training standards are met:

‘I think it needs to be done based on an agreed minimum standard for one to be called a physiotherapist; you need to say what should be covered.’ (Transcript 5)

‘So I think policy, that’s the word, has also impacted the role of clinicians.’ (Transcript 14)

Clinical governance ensures high-quality, safe, effective patient care and includes patient satisfaction (Bishop 2008). The key to clinical governance lies in good clinical education as this offers the framework to maintain minimum standards and evidence-based practice. This makes the relationship between clinicians, clinical educators and physiotherapy students very important, and this relationship featured strongly in the FGDs:

‘One thing I specifically remember as a student was that a lot depended on the clinician that you worked with. They could either make you or break you.’ (Transcript 6)

### Structure

The structure (meso level) includes the physical and academic organisational aspects of the various clinical settings and the academic organisation of clinical experiences for students. The academic organisation consists of the multiple types of clinical settings; the number of clinical experiences; timing and duration of these experiences – the length, breadth and depth of these exposures; the sequencing of the exposures; supervisory models and the experience and skills of the clinical educators (Jette et al. 2014; McCallum et al. 2013).

‘My first thought that comes to mind is exposure to the right variety of conditions and situations and patients.’ (Transcript 5)

‘I think that for me in the broad sense, the very broad sense it’s the choice of placements. There must be a variety to tap into to expose students to all the differences.’ (Transcript 10)

In addition, the processes of supervision, evaluation and the underlying administrative procedures that rely on structures and resources further impact the clinical learning experience (Jette et al. 2014; Strohschein et al. 2002).

‘Part of the structure is how it is coordinated between the [*academic*] departments and the various clinical facets. So, any clinical support strategy to make sure the programme is run smoothly, and that communication is clear between the hospitals and the university [*is important*].’ (Transcript 5)

‘The university and the Minister of Education looks at throughputs. So, you know your programme is effective.’ (Transcript 12)

### Clinical learning experience

The micro level included the clinical learning experience itself. The clinical learning experience impacts graduates and should be a key consideration in any clinical curriculum. Participants had strong statements about their experiences:

‘I was mentored by my clinicians when I was a student. There was an obvious sense that they took me under their wing and they mentored me through my clinical placements with them.’ (Transcript 14)

The factors that influence the clinical learning experience include the student-supervisor relationship, teaching skills and methods, feedback, characters of the students and supervisors, resources, institutional culture and the atmosphere of the learning environment (Dolmans et al. 2008; Ernstzen & Bitzer 2009; Ernstzen, Bitzer & Grimmer-Somers 2010; Maloney, Stagnitti & Schoo 2013; Meyer, Louw & Ernstzen 2019; Newton, Billett & Ockerby 2009; Odole et al. 2014).

‘I think it’s important that when you see that your student is very comfortable with a specific type of condition, challenge them-take them out of their comfort zone so that they can also learn how to be comfortable with a different environment or a different condition.’ (Transcript 4)

Patton et al. (2018) have disputed the student-supervisor relationship mentioned in the studies earlier. They show how four key factors have a symbiotic relationship: workplace influences, professional practice engagement, supervisors’ intentions and students’ dispositions and experiences. They describe these as fluid in nature, and the ability of these factors to influence clinical learning depends on any current situation, thus not one of these factors is superior to another. These powerful learning influences confirm the complex clinical space that students need to navigate to gain the unique experiential learning that is offered by clinical education (Patton et al. 2018):

‘[*N*]eeds to be a friendly environment where the students can feel comfortable but it also needs to be an open environment … Students must know that the supervisor will help if they have a problem.’ (Transcript 1)

These emerging themes are specific to the South African situation, which is a limitation of our study. The resultant tool would have to undergo some changes to be universally acceptable.

## Conclusion

Items were generated from codes under each theme, resulting in the preliminary tool. The themes and the extensive number of items generated (131 items) reflect the complexity and diversity of physiotherapy clinical education currently navigated. This first step in developing a tool that pulls together and encapsulates the breadth and depth of physiotherapy clinical education challenged several scholars. The items in these themes can now be considered for the next stage of the development of a tool to evaluate clinical physiotherapy education.

### Implications for physiotherapy practice

Introducing a tool to evaluate physiotherapy clinical education may ensure the minimal standard for clinical physiotherapy education and quality assurance processes.
